# Needle-knife therapy improves the clinical symptoms of knee osteoarthritis by inhibiting the expression of inflammatory cytokines

**DOI:** 10.3892/etm.2014.1516

**Published:** 2014-01-30

**Authors:** MUNAN LIN, XIHAI LI, WENNA LIANG, JIANHUA LIU, JIANHONG GUO, JINGXIONG ZHENG, XIANXIANG LIU

**Affiliations:** 1 Department of Traditional Chinese Medicine, Fuzhou General Hospital of Nanjing Military Command, Fuzhou, Fujian 350025, P.R. China; 2 Academy of Integrative Medicine, Fujian University of Traditional Chinese Medicine, Fuzhou, Fujian 350108, P.R. China; 3 Research Base of Traditional Chinese Medicine Syndrome, Fujian University of Traditional Chinese Medicine, Fuzhou, Fujian 350122, P.R. China

**Keywords:** needle-knife therapy, osteoarthritis, knee joint, acupuncture

## Abstract

Knee osteoarthritis (KOA) is a degenerative joint disease that occurs mainly in the elderly population. However, there are currently no effective treatments for treating this condition. In this study, the efficacy of needle-knife therapy, a technique of traditional Chinese medicine that has been widely used to treat KOA was investigated. Patients (n=170) with KOA were randomly divided for needle-knife therapy (treatment group) and acupuncture therapy (control group). Outcome evaluation included stiffness, pain, physiological function, overall changes, total symptom score, clinical curative effects and the concentrations of interleukin (IL)-1β, IL-6 and tumor necrosis factor-α (TNF-α) in the synovial fluid. The trial was completed in 151 patients (233 knees) from a total of 170 patients (264 knees); the treatment group comprised 76 patients (117 knees) who completed the trial and 9 patients (14 knees) who were removed from the study, and the control group comprised 75 patients (116 knees) who completed the trial and 10 patients (17 knees) who were removed from the study. The symptom scores of KOA in stages I–IV were reduced significantly in the treatment group and those of stages I–III were decreased significantly in the control group. The effective rate of the KOA therapy in the patients of stages III and IV in the treatment group was significantly higher than that in the control group. After treatment, the decrements of IL-1β, IL-6 and TNF-α in the treatment group were greater than those in the control group. These results showed that the use of needle-knife therapy to treat KOA effectively improved the clinical symptoms by inhibiting the expression of inflammatory cytokines.

## Introduction

Knee osteoarthritis (KOA) is a degenerative disorder that results from cartilage failure or an imbalance of the degradation and repair processes of chondrocytes, cartilage matrix and subchondral bone, which are induced by a complex interplay of biochemical and biomechanical factors with secondary components of inflammation (1,2). Studies have shown that the incidence of KOA in people aged >65 years ranges from 60 to 70%, with the incidence rate reaching up to 85% in the population aged >75 years (3). Joint stiffness and pain are the main clinical symptoms of KOA (4). However, there is no effective treatment for KOA. Care focuses on alleviating the symptoms of stiffness and pain, and improving or maintaining the physical function of the knee joint, based on the guidelines published by the American College of Rheumatology (5,6).

Conventional therapies for KOA, which are designed to regulate the symptoms of stiffness and pain, include topical analgesics, glucosamine, nonsteroidal anti-inflammatory drugs, intra-articular injection of sodium hyaluronate and surgical treatment (7,8). However, these therapies are not considered curative, and are usually accompanied by a number of side-effects ranging from patient discomfort to kidney and liver damage. The majority of patients with KOA are not satisfied with the recurring side-effects of conventional drug therapies, which results in the use of alternative and complementary therapies (9,10). Traditional Chinese medicine (TCM), including acupuncture and Chinese herbal medicine, has been shown to be particularly efficacious for treating pain for thousands of years, especially when related to joint diseases such as KOA (11–13). At present, an increasing number of patients are turning to alternative and complementary medicine owning to the limitations and side-effects of conventional therapies. Therefore, it is necessary to explore the mechanisms of these therapies for treating KOA.

KOA is a chronic, retrogressive knee disease often occurring in the elderly, with most cases belonging to the TCM ‘stasis of the channels’ type (14). In needle-knife therapy, a physician stimulates local tissue using a needle-knife to loosen and release adhesions so as to relieve tension pain and recover internal force equilibrium of the knee joint (15). Needle-knife therapy may also slow down the degradation of articular cartilage and improve and maintain the function of the joint (16). Indications of KOA include joint and myofascial pain, and refractory pain and inflammation in rheumatoid disorders. Due to the fact that it causes minimal injury to local tissue, does not compromise the overall structure of the knee and enables rapid patient recovery, needle-knife therapy has been widely used to treat a variety of rheumatoid disorders involving joint stiffness, pain and swelling, including rheumatoid arthritis and KOA (15,17,18).

The aim of the present study was to identify a more effective therapy for reducing joint pain and disability, as well as to prevent and mitigate cartilage degradation. A randomized, controlled study was performed to evaluate the safety and efficacy of needle-knife therapy for treating KOA. The basic design of this study involved the quantification and comparison of the efficacy of needle-knife therapy and acupuncture for KOA. The effects of the two treatments on the clinical symptoms of KOA and the expression of inflammatory cytokines were investigated.

## Materials and methods

### Patient information

A total of 170 patients with KOA from the Fuzhou General Hospital of Nanjing Military Command (Fuzhou, China) from April 2010 to March 2013 were enrolled in this study. The clinical characteristics of the patients were recorded in a uniform data collection table.

All patients enrolled were randomly assigned to two groups by lottery and their general characteristics are listed in Table I. There was no significant difference in gender, age, illness course and number of affected knees between the two groups (P>0.05); hence, the groups were comparable. The study was approved by the Ethics Committee of the Fuzhou General Hospital of Nanjing Military Command. Informed consent was obtained from all patients.

### Standards for diagnosis and inclusion

#### Standards for the diagnosis of KOA in Western medicine

The diagnosis of KOA was formulated according to the standards issued by the American College of Rheumatology (5,19). Patients must have either knee joint pain or osteophytes and fulfill at least one of the following three criteria: i) Age >40 years; ii) morning stiffness lasting <30 min and an audible sound of bone friction; and iii) enlarged tender bone and no evident heat in the joint. The severity of KOA was classified into 5 stages (20), specifically: Stage 0, normality; stage I, appearance of lip-like osteophytes; stage II, noticeable osteophytes narrowing the joint gap; stage III, moderate and multiple osteophytes markedly narrowing the joint gap with bony sclerosis and wear; and stage IV, large osteophytes markedly narrowing the joint gap with serious bony sclerosis and evident wear of the bone.

#### Standards for the diagnosis of KOA in TCM

The TCM syndrome of ‘stasis of the channels’ type was differentiated with reference to the standards for diagnosis in Efficacy Evaluation of TCM Diseases and Syndromes and the Guiding Principle of Clinical Research on New Drugs of Traditional Chinese Medicine (14). Diagnosis included knee joint pain, difficulty in flexion and extension, weakness and soreness in the loin and knees, accompanied by the presence of a reddish tongue with a thin or thin greasy coating and a taut pulse.

### Standards for exclusion

The patients with the following conditions were excluded: Patients aged >70 years; patients with concurrent rheumatoid arthritis, psoriasis, syphilitic neuropathy, ochronosis, metabolic osteopathy, acute trauma and other diseases affecting the joints; women in pregnancy or lactation; patients with accompanying severe cardiovascular, hepatic, renal or with mental disease; patients who had been treated with other methods that may have influenced the observation of indices in this study; and patients who discontinued treatment during the study or refused to objectively provide evaluation data in this study.

### Treatment

**Figure 1:**
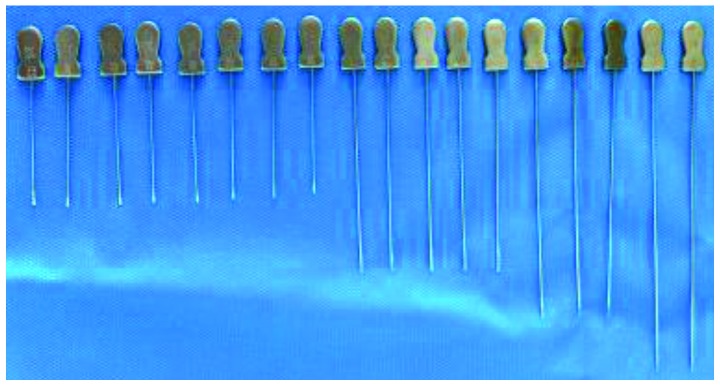
Needle-knife instruments.

**Figure 2:**
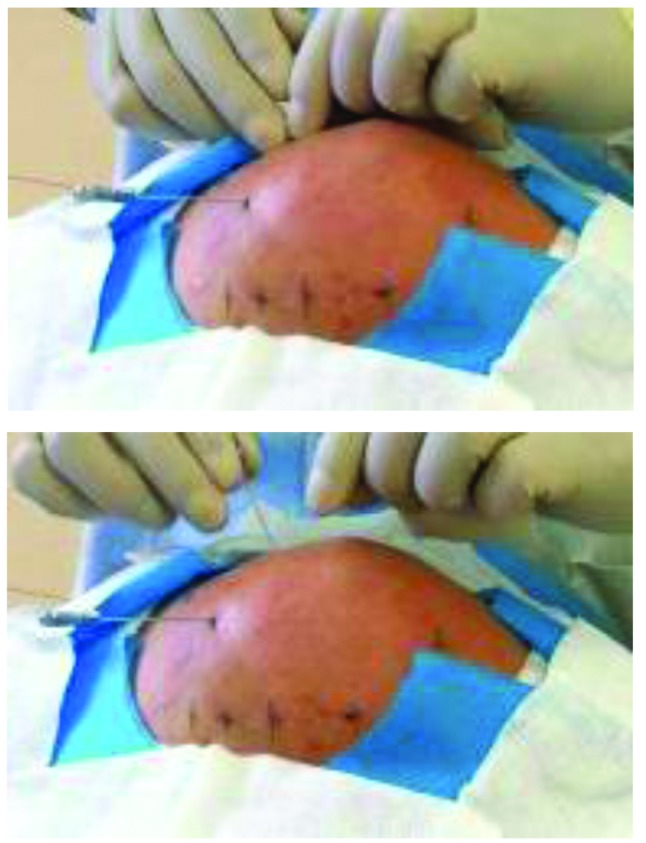
Therapeutic process of needle-knife treatment, including selecting acupoints (upper image) and needle-knife insertion (lower image).

The patients in the treatment group were treated with needle-knife (Hanzhang Acupotome; Beijing Huaxia Acupotome Medical Equipment Factory, Beijing, China) therapy at the dominant acupoints of Neixiyan (Ex-LE4) and Waixiyan (Ex-LE5), as well as the conjugate points Xuanzhong (GB39), Xuehai (SP10), Dubi (ST35) and Taixi (KI3) (Figs. 1 and 2). The patient lay in a supine position with general skin disinfection. After the acupoints were disinfected, needle-knives was inserted at the dominant acupoints, parallel to the direction of muscles, nerves and vessels. Following the needle-knife surgery, patients undertook passive activities of knee flexion, extension and rotation. The treatment was conducted once as one therapeutic course and two courses were administered to each patient with a 6-day interval between the two courses.

The patients in the control group were treated with acupuncture at the dominant acupoints of Neixiyan and Waixiyan, as well as the conjugate points Xuanzhong, Xuehai, Dubi and Taixi. The patients were seated with the knee joints flexed. After the acupoints were disinfected, routine, disposable needles were inserted at the dominant acupoints for 1.5 Cun, with twisting up and down to induce ‘De-Qi’. The treatment was conducted daily for 5 days as one therapeutic course, and two courses were administered to each patient with a 2-day interval between the two courses.

### Indices for observation

The clinical symptoms scores of the patients were evaluated for 11 items following the criteria set in reference to the Guiding Principle of Clinical Research on New Drugs of TCM (14): i) Morning pain or stiffness after getting up; ii) resting pain of the knee joint; iii) sensation of discomfort or pain while walking; iv) swollen knee joint; v) tenderness of the knee joint; vi) difficulty in extension and flexion of the knee joint; vii) maximum distance of walking; viii) daily life activity; ix) heat sensation in the local skin of the knee joint; x) local reddish skin; and xi) help needed when moving from a sitting to a standing position.

The symptoms of items i)–vii) were ranked by severity in three grades and scored from 1 to 3; the more severe the symptom, the higher the score, and no symptom scored 0. A similar method was performed on the symptoms of items viii) and ix), but they were ranked in two grades and scored from 1 to 2; as for items x) and xi), when they occurred, the score was 1, otherwise it was 0. The severity of KOA was estimated in three grades based on the total score of symptoms (the sum of various symptoms): mild grade, the total score was <10; moderate grade, the total score was from 10 to 18; and severe grade, the total score was >18. The maximum possible symptom score was 33.

### Standard for curative effects

With reference to the Guiding Principle of clinical Research on New Drugs of TCM (14), according to the subjective sensation of the patients and their knee joint function, the therapeutic efficacy on the affected knee joints was evaluated using the following four grades: Grade 1 (excellent), symptoms disappeared with normal function and the severity of KOA scored 0 or 1; grade 2 (good), symptoms basically disappeared and function was recovered, enabling daily activities and work to be carried out, and the KOA severity score was decreased by >2/3; grade 3 (moderate), pain disappeared with basic normal knee joint extension and flexion functions, some improvement in daily activities, and the KOA severity score was reduced by between 1/3 and 2/3; and grade 4 (bad), no evident alleviation of the symptoms. The percentage of knees in a group that were of the former three grades was defined as the effective rate.

### Determination of inflammatory cytokine levels

For the assessment of the IL-1β, IL-6 and TNF-α levels in the synovial fluid, the synovial fluid collected from the patients with KOA prior to and following treatment was dispensed into 1-ml aliquots. The synovial fluid was diluted with diluent’s buffer (Nanjing Jiancheng Biochemicals Ltd., Co., Nanjing, China) to the appropriate detection range for evaluation by enzyme-linked immunosorbent assay (ELISA). The levels of the proteins of interest in the synovial fluid were measured using commercially available ELISA kits (Nanjing Jiancheng Biochemicals Ltd., Co.), following the manufacturer’s instructions.

### Safety evaluation

The symptoms and physical signs of adverse reactions that occurred following the two treatments (needle-knife therapy and acupuncture) were recorded, and routine blood, urine and stool tests, electrocardiogram (ECG) tests, and evaluations of liver and renal functions were performed before and after treatment.

### Statistical analysis

All data were analyzed using SPSS software for Windows, version 13.0 (SPSS, Inc., Chicago, IL, USA). Statistical analysis of the data was performed with Student’s t-test and one-way analysis of variance. The enumeration data was analyzed by the chi-square test. P<0.05 was considered to indicate a statistically significant difference.

## Results

### Accomplishment of study

From the 170 patients (264 knees) enrolled in this study, 151 patients (233 knees) completed the trial and 19 patients (31 knees) were removed. In this study, 76 patients (117 knees) and 75 patients (116 knees) completed the trial in the treatment and control groups, respectively. The nineteen patients (31 knees) that were excluded due to uncompleted prescribed therapeutic courses included nine patients (14 knees) in the treatment group with two joints of stage I, three joints of stage II, five joints of stage III and four joints of stage IV; and 10 patients (17 knees) in the control group, which comprised three joints of stage I, six joints of stage II, five joints of stage III and three joints of stage IV. The exclusion rates in the two groups were not significantly different and the influence of exclusion on the intergroup proportionality of the baseline was negligible.

### Comparison of total symptom scores of KOA

**Figure 3:**
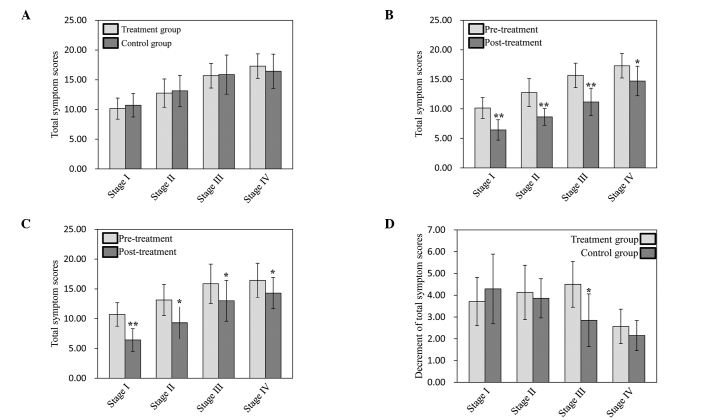
Comparison of the total symptom scores of KOA. Total symptom scores of KOA (A) prior to treatment in the treatment and control groups, (B) prior to and following treatment in the treatment group and (C) prior to and following treatment in the control group; and (D) decrement of total symptom scores of KOA following treatment in the treatment and control groups. Data are expressed as the means ± SD and the SD is shown as a vertical bar. (B and C) ^*^P<0.05, ^**^P<0.01, compared with the value prior to treatment in the same group; (D) ^*^P<0.05, compared with the decrement of total symptom scores in the control group. KOA, knee osteoarthritis; SD, standard deviation.

The symptom scores of KOA at the corresponding stages were not significantly different between the two groups prior to treatment (P>0.05). Following treatment, the symptom scores of KOA in stages I–IV were reduced significantly in the treatment group (P<0.05 or P<0.01), and the scores of KOA in stages I–III were decreased significantly in the control group (P<0.05 or P<0.01), while those of stage IV were not significantly changed (P>0.05), although a slight reduction was detected (Fig. 3A–C).

In a comparison between the two groups, with the exception of a significant reduction in the total symptom scores of KOA in stage III in the treatment group compared with that in the control group (P<0.05), no significant differences were observed in the symptom scores of KOA at other stages of the disease (P>0.05), although a few divergences were revealed (Fig. 3D).

### Comparison of clinical effectiveness

No statistically significant difference between the two groups was detected in the excellent rates of the treatments in the patients at stages I–IV and in the effective rates of the treatments in the patients of stages I and II (P>0.05). The effective rate in patients of stages III and IV in the treatment group was significantly higher than that in the control group (P<0.05) (Table II).

### Comparison of the synovial fluid concentrations of IL-1β, IL-6 and TNF-α

**Figure 4:**
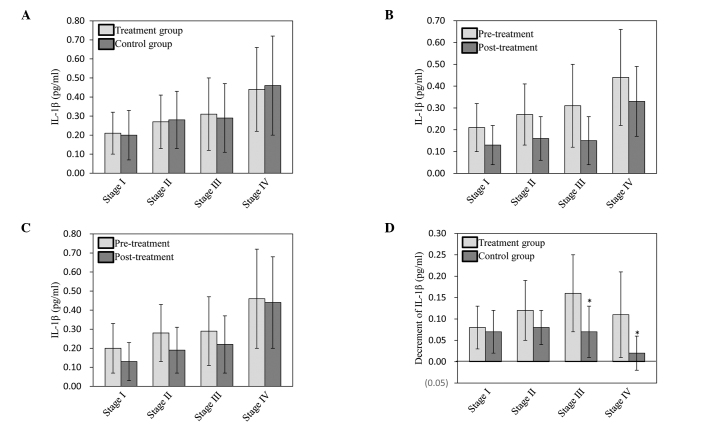
IL-1β concentrations in the synovial fluid. IL-1β concentrations in patients with KOA (A) prior to treatment in the treatment and control groups; (B) prior to and following treatment in the treatment group and (C) prior to and following treatment in the control group; and (D) decrement of IL-1β concentrations following treatment in the treatment and control groups. Data are expressed as the means ± SD and the SD is shown as a vertical bar. ^*^P<0.05, compared with thedecrement of IL-1β in the control group. IL, interleukin; KOA, knee osteoarthritis; SD, standard deviation.

**Figure 5:**
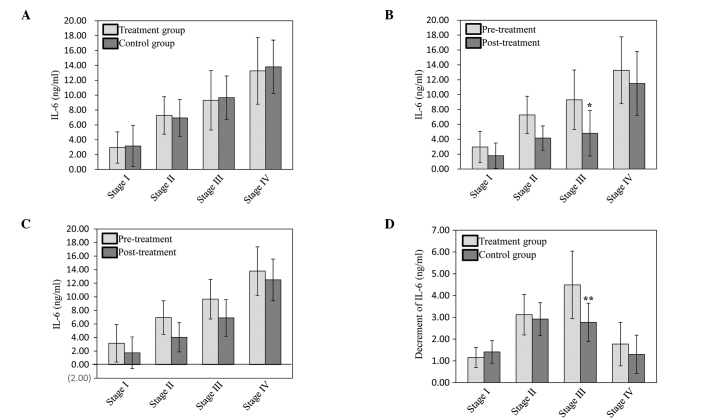
IL-6 concentrations in the synovial fluid. IL-6 concentrations in patients with KOA (A) prior to treatment in the treatment and control groups; (B) prior to and following treatment in the treatment group and (C) prior to and following treatment in the control group; and (D) decrement of IL-6 concentrations following treatment in the treatment and control groups. Data are expressed as the means ± SD and the SD is shown as a vertical bar. ^*^P<0.05, compared with prior to treatment in the same group; ^**^P<0.01, compared with the decrement of IL-6 in the control group. IL, interleukin; KOA, knee osteoarthritis; SD, standard deviation.

**Figure 6:**
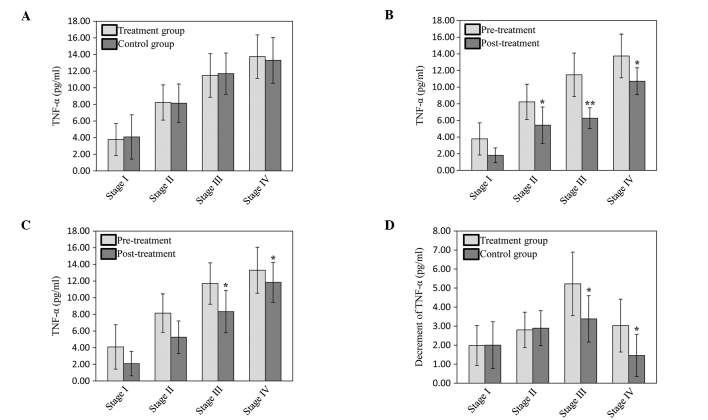
TNF-α concentrations in the synovial fluid. TNF-α concentrations in patients with KOA (A) prior to treatment in the treatment and control groups; (B) prior to and following treatment in the treatment group and (C) prior to and following treatment in the control group; and (D) decrement of TNF-α concentrations following treatment in the treatment and control groups. Data are expressed as the means ± SD and the SD is shown as a vertical bar. (B and C) ^*^P<0.05, ^**^P<0.01, compared with prior to treatment in the same group; (D) ^*^P<0.05, compared with the decrement of TNF-α in the control group. IL, interleukin; KOA, knee osteoarthritis; SD, standard deviation.

To evaluate the effect of needle-knife therapy on the expression levels of inflammatory cytokines, the concentrations of IL-1β, IL-6 and TNF-α in the synovial fluid of the patients with KOA were investigated by ELISA. As shown in Figs. 4–6, the synovial fluid concentrations of IL-1β, IL-6 and TNF-α were not different between the treatment and control groups. In stages III and IV of KOA, the decrement of IL-1β in the treatment group was significantly higher than that in the control group (P<0.05). In stage III of KOA, the synovial fluid concentration of IL-6 in the treatment group was significantly different between pre- and post-treatment (P<0.05), and the decrement of IL-6 in the treatment group was significantly decreased compared with that of the control group (P<0.01). The concentration of TNF-α in the synovial fluid following treatment was markedly decreased compared with that prior to treatment in stages II, III and IV of the treatment group (P<0.05 and P<0.01), while the changes of TNF-α levels in the control group in stages III and IV were similar to those in the treatment group (P<0.05). This suggests that needle-knife therapy inhibited the expression of inflammatory cytokines.

### Comparison of X-ray features

No significant changes in the X-ray features between before and after treatment were observed in either group, indicating that although treatment in the two groups effectively alleviated the symptoms of KOA, it did not improve the organic changes that had previously occurred.

### Safety evaluation

No adverse reaction-associated signs and symptoms or laboratory indices were identified during the whole treatment course.

## Discussion

The results of the present support the use of needle-knife therapy as a safe and effective method for the treatment of KOA. It was shown to be more effective than routine acupuncture for the alleviation of pain and improvement of physiological function by inhibiting the expression of inflammatory cytokines. In addition, the results also indicated that acupuncture may relieve knee stiffness and pain and improve the function scores of KOA.

KOA increases in prevalence with age and is a major cause of pain and locomotor disability worldwide. It belongs to the category of Gu Bi in TCM, which means either the limbs or the joints are suffering from pain and malfunction. TCM suggests that the disease is based on Gan-Shen insufficiency and weakness of tendons and bones, and is importantly linked to blood stasis (21). Currently, there is no definitive cure for KOA, and available treatments are aimed at improving pain and function in the hopes of delaying knee replacement surgery (22). TCM has shown significant advancements against KOA, such as improving the clinical presentation of patients, and inhibiting inflammatory reaction and cartilage degradation (23). Needle-knife, an ancient traditional Chinese medical therapy, is used widely to treat KOA. When practiced by a certified provider, it is safe and patients often find it calming and relaxing. Although the biological mechanisms by which needle-knife therapy improves the clinical consequences of KOA are not fully understood, its analgesic effect and ability to improve cold-dampness pathogen components are likely to have an important role (24). In order to observe the curative effects of needle-knife therapy for treating KOA, the curative effects and changes in the total score of symptoms prior to and following treatment between a needle-knife therapy group and an acupuncture therapy group were determined.

Patients with KOA require better pain control and reduced adverse events. Therefore, as comorbidities and aging increase, a more convenient approach is necessary (25). Needle-knife, as an effective, safe and non-pharmaceutical therapy regimen, has also been used widely for the management of KOA. According to TCM theory, KOA is a bone obstruction disease, in which the knee joints suffer from stiffness, pain, and/or malfunction due to invasions of dampness or wind cold, accompanied by the disharmony of Qi and blood, consequently resulting in the syndrome of ‘blood stasis’ and ‘cold dampness’ (26,27). Therefore, the acupoints of Neixiyan (Ex-LE4), Waixiyan (Ex-LE5), Xuehai (SP10) and Dubi (ST35) were selected in the treatment for activating the blood, resolving stasis, dispelling cold and removing dampness, which may induce stimulation, directly reaching the illness site and acting to alleviate clinical symptoms and improve the function of the knee joint.

According to the features of KOA, two widely applied acupoints, Xuanzhong (GB39) and Taixi (KI3), were selected to dredge the meridian-collaterals and nourish Shen to strengthen bone, which may effectively improve the Gan-Shen insufficiency and weakness of tendons and bones, raise the endurance of peri-articular tissue to inflammatory stimulation, and be helpful for knee joint pain alleviation and knee joint function restoration to attain good therapeutic effectiveness. Hence, needle-knife therapy may effectively alleviate pain in the knee joints and improve the scope of motion. Results of this study showed that the clinical symptoms in patients with KOA of stages I–IV were improved significantly following treatment with needle-knife or acupuncture therapy, while needle-knife therapy showed a better efficacy than acupuncture for the patients of stages III and IV.

KOA is characterized by an imbalance of matrix synthesis and matrix degradation in cartilage at the cell and tissue levels. Chondrocytes, the only cell type present in articular cartilage, are responsible for the synthesis and breakdown of the extracellular matrix (ECM) (28). Signals generated by growth factors, cytokines and the ECM control chondrocyte metabolic activity. During the pathological progression of KOA, excessive ECM degradation overwhelms ECM synthesis and this appears to be due to inflammatory and catabolic signals that are present in excess of the anti-inflammatory and anabolic signals. Pro-inflammatory cytokines associated with KOA include IL-1β, IL-6 and TNF-α (29). Of these cytokines, IL-1β is considered to be the major cytokine mediating cartilage destruction, which induces a cascade of inflammatory and catabolic events, including the expression of nitric oxygen production, prostaglandin E2 release and cartilage degrading matrix metalloproteinases, while inhibiting collagen and proteoglycan synthesis (30–33). In addition, IL-6 is thought to have a regulatory role and is capable of downregulating type II collagen gene expression in articular chondrocytes (34). TNF-α and IL-1β also affect the synthetic activity of chondrocytes by inhibiting the synthesis of type II collagen and proteoglycans (35,36). The results of the present study showed that the decrements of IL-1β, IL-6 and TNF-α in the treatment group were greater than those in the control group.

The preliminary findings in the present study indicate that needle-knife therapy is effective in improving knee pain, stiffness and physical function in patients with KOA. The curative and the long-term curative effects are to be determined in further follow-up visits. The major limitation in the present study was the small sample size, and a randomized controlled study with a larger sample size will be conducted in the future.

## Figures and Tables

**Table I:** General characteristics of patients with knee osteoarthritis.

Group	Cases	Affected knee (cases)		Stage	Gender (cases, M/F)	Age (years)	Course of disease (months)	No. of knees

		Single	Bilateral					
Treatment	85	39	46	I	9/11	52.52±8.63	10.76±7.92	28
				II	13/10	57.66±11.35	18.34±11.47	35
				III	11/14	58.71±10.54	24.22±15.24	47
				IV	8/9	60.43±7.87	26.92±10.53	21
Control	85	37	48	I	8/10	51.43±9.12	11.36±8.41	26
				II	12/14	55.94±12.67	16.94±13.62	44
				III	12/13	59.36±13.50	22.53±17.72	43
				IV	9/7	61.65±8.92	27.83±11.42	20

**Table II:** Comparison of clinical effectiveness.

Group	Cases	Stage	No. of knee	Grade of effectiveness (no. of knee)				Excellent rate (%)	Effective rate (%)

				Excellent	Good	Moderate	Bad		
Treatment	76	I	26	5	12	6	3	65.38	88.46
		II	32	3	12	11	6	46.88	81.25
		III	42	0	7	26	9	16.67	78.57*
		IV	17	0	1	10	6	5.88	64.71*
Control	75	I	23	3	11	7	2	60.87	91.30
		II	38	2	15	13	8	44.74	78.95
		III	38	0	4	19	15	10.53	60.53
		IV	17	0	1	6	10	5.88	41.18

* P<0.05, compared with the effective rate in the control group.
